# Ethanolic Fermentation of Rye Mashes: Factors Influencing the Formation of Aldehydes and Process Efficiency

**DOI:** 10.3390/biom12081085

**Published:** 2022-08-06

**Authors:** Katarzyna Pielech-Przybylska, Maria Balcerek, Maciej Klebeko, Urszula Dziekońska-Kubczak, Mariusz Hebdzyński

**Affiliations:** Faculty of Biotechnology and Food Sciences, Institute of Fermentation Technology and Microbiology, Lodz University of Technology, Wolczanska 171/173, 90-530 Lodz, Poland

**Keywords:** starchy raw materials, distillery mash, fermentation, yeast, by-products, aldehydes

## Abstract

High concentrations of aldehydes may result in poor-quality agricultural distillate. We investigate the influence of the method of mash preparation, the initial pH of the mashes, and different yeast strains on the fermentation efficiency and concentration of aldehydes from C2 (acetaldehyde) to C7 (enanthaldehyde) in rye mashes. The tested factors were revealed to have a differentiated influence on both the process efficiency and the concentrations of aldehydes, especially in the case of the dominant acetaldehyde. Mashes obtained from steamed rye grain showed significantly higher fermentation efficiencies than those prepared by the pressureless method. Increasing the pH of the sweet mashes from 4.5 to 6.0 resulted in significantly higher concentrations of acetaldehyde, especially in the case of steamed rye grain. Moreover, an increase in the concentrations of other aldehydes, i.e., from C3 (propionaldehyde) to C5 (valer- and isovaleraldehyde) was observed. A high fermentation efficiency and the lowest acetaldehyde concentrations were obtained from steamed rye mashes with an initial pH of 4.5, fermented using the yeast strains DistilaMax GW and DistilaMax HT. DistilaMax HT yeast also provided a relatively low concentration of acetaldehyde in mashes with an initial pH in the range of 4.5–5.5 prepared by the energy-saving pressureless method.

## 1. Introduction

A distillate of agricultural origin, known as a raw spirit, is an alcoholic liquid obtained by the alcoholic fermentation and subsequent distillation of agricultural products listed in Annex I to regulation 2019/787 of the European Parliament and of the Council of 17 April 2019 [1]. A raw spirit does not have the properties of either ethyl alcohol or a spirit drink but retains the aroma and taste of the raw materials used. The quality of agricultural distillates depends on the qualitative and quantitative composition of volatile compounds. Several factors have a substantial influence on the chemical composition of agricultural distillates, such as the type and quality of the used starchy raw materials, the method of starch liberation and mashing, the yeast strains used, the conditions of fermentation (pH, temperature), microbial contamination, and the distillation process [2].

A number of volatile fermentation by-products are present in raw spirits, including carbonyl compounds, alcohols, esters, acids, and acetals. These compounds are present in a very wide range of concentrations. Some have a beneficial influence on the sensory features of spirit beverages, such as whisky, Starka, or Kornbrand. Others have a negative impact, even when present only in trace amounts [3]. Further processing of low-quality distillates is expensive, causing financial losses to the manufacturer [4].

Carbonyl compounds, especially aldehydes, have often been noted to have a negative influence on the quality of spirits. Aldehydes may be formed by the degradation of amino acids, oxidation of alcohols, or autoxidation of fatty acids [5]. These reactions can occur during the thermal treatment of starchy raw materials [6], fermentation, distillation, or storage. A high content of aldehydes (mainly acetaldehyde and acrolein) in spirit beverages may also result from microbial contamination of distillery mashes [7].

Aldehydes contained in alcoholic beverages are intermediates of the two-step processes of α-keto acid decarboxylation to alcohols [8]. Several enzymes have been identified as being involved in the catabolism of α-keto acids derived from aromatic and branched-chain amino acids in *Saccharomyces cerevisiae* yeast [9,10]. Apart from requiring fermentable carbohydrates, for optimal alcohol production, yeast fermentation should be supplemented with appropriate nutrients, including sufficient nitrogen, minerals, vitamins, and oxygen [10,11]. During a properly conducted process, the concentration of acetaldehyde rises in the early phase of fermentation, but then falls in the stage of turbulent phase, when acetaldehyde is reduced to ethanol [12]. An increased concentration of acetaldehyde can also result from alterations to the parameters of the fermentation procedure, such as oxygenation of the medium, pH, the concentration of fermenting sugars, yeast strain, inoculum size, and process temperature [13,14]. The presence of inhibitors of enzymatic reactions can result in the production of higher aldehydes, which are produced along metabolic pathways leading to higher alcohols [15,16].

Acetaldehyde is the most abundant aldehyde in spirits. Although characterised by a fruity odour, at higher concentrations, it causes a very sharp and unpleasant taste. Other longer chain aldehydes present in alcoholic beverages, including distillates, also produce a stinging sensation described as “trigeminal burn” [17]. The sensorial perception threshold has been found to decrease exponentially with the chain length of the compound [18,19]. Acetaldehyde is a group 2B carcinogen, whereas acetaldehyde obtained by the consumption of alcoholic beverages is classified as a group 1 carcinogen [20]. According to the Polish Standard [21], the concentration of acetaldehyde in agricultural distillates should not exceed 100 mg/L of absolute alcohol. The current EU regulation [1] does not set any limits on the acetaldehyde content in agricultural distillates.

The purpose of this study was to investigate the concentrations of aldehydes in distillery mashes, taking into account the following variables: methods of distillery mash preparation (pressure-thermal and pressureless); the initial pH of the mash (between 4.5 and 6.0 units); and three yeast strains recommended for spirit beverages or ethyl alcohol of agricultural origin. The qualitative and quantitative composition of the sugars, ethanol, and other compounds in the mashes were determined before and after fermentation to evaluate process efficiency.

## 2. Materials and Methods

### 2.1. Materials

The following materials were used in the study:

- Rye grains of the Amilo cultivar (Danko Plant Breeding Ltd., Choryń, Poland).
- Enzyme preparations (Novozymes A/S, Bagsværd, Denmark): Termamyl SC (α-amylase) for starch liquefaction at a dose of 0.13 mL per 1 kg of starch; SAN Extra (glucan 1,4-α-glucosidase) for starch saccharification at a dose of 0.6 mL per 1 kg of starch; Viscoferm**^®^** (a multienzyme complex containing non-starch-degrading enzymes) at a dose of 0.15 mL per 1 kg of raw material; Neutrase (protease) at a dose of 0.15 mL per 1 kg of raw material.
- Dry distillery yeast strains (*Saccharomyces cerevisiae*), all at a dose of 0.5 g d.m./L of mash: DistilaMax HT (Lallemand Inc., Montréal, QC, Canada), which is thermotolerant and a low producer of congeners, recommended for use in the production of vodka, neutral spirits, and light flavoured beverages; DistilaMax GW (Lallemand Inc., Canada), which is recommended for use in the production of American-style whiskies from various whole grains; Ethanol Red (Fermentis Division of S.I. Lesaffre, Marcq-en-Barœul, France), which is recommended for the production of industrial ethanol from starchy substrates.

### 2.2. Sweet Mash Preparation

Sweet mashes were prepared using two methods:

- The pressureless starch liberation (PLS) method. Milled rye grain was mixed with tap water at a ratio of 3.5 L water per 1 kg of milled grain in a vessel placed in a water bath and equipped with a laboratory stirrer and thermometer. The mixture was continually stirred and heated to 50 °C. A liquefying Termamyl SC α-amylase preparation and a viscosity reducing Viscoferm**^®^** preparation were added. The mixture was heated to 90 °C with continuous stirring. It was kept for 60 min at this temperature, then cooled to approximately 65 °C, digested with a saccharifying SAN Extra preparation and supportive Neutrase preparation and kept for 30 min at 65–50 °C. The mash was then cooled to a temperature of 30 °C (optimal for yeast inoculation). Its pH was adjusted with a sulfuric acid solution (25% *w*/*w* ) to 4.5, 5.0, 5.5, and 6.0, and supplemented with an aqueous solution of (NH_4_)_2_HPO_4_ (0.2 g/L mash) as a nutrient for yeast.
- The pressure-thermal method. Rye grain (5 kg) was placed in a tapered cylindrical steamer previously filled with 17.5 L of water heated to boiling point. The steamer was then closed. The raw material was steamed at 150 °C with a pressure of 0.4 MPa for 35 min, with periodical circulation of the content. The content of the steamer was subsequently transferred to a cylindrical steel mashing vessel equipped with a heating/cooling coil and a thermometer. The mashing process was the same as in the PLS method.

### 2.3. Fermentation of Mashes

Before inoculation of the rye mashes, an appropriate amount of yeast was hydrated and disinfected (15 min incubation of cells suspended in 25% *w*/*w* sulfuric acid solution, pH 2.5, at room temperature to eliminate weaker yeast cells and unintended bacterial cells). The obtained yeast cream was added to the mash (without neutralisation). The inoculated mashes were mixed carefully. Fermentation was conducted for 3 days at 30 °C in 0.5 L glass bottles containing 0.25 L of mash. The bottles were closed with fermentation locks containing paraffin oil. The process was controlled gravimetrically.

### 2.4. Analytical Methods

Distillery mashes were analysed in terms of quality assessment parameters. The total extract, i.e., the concentration of dissolved solids (mostly sugar) in the sweet mashes, was measured using an areometer with a scale in g/kg [22]. The quantitative and qualitative composition of the sugars (before and after acid hydrolysis of starch), as well as of ethanol and fermentation by-products in the distillery mashes (before and after fermentation), were determined by the HPLC technique, using an Infinity 1260 liquid chromatograph (Agilent Technologies, Santa Clara, CA, USA) equipped with a refractive index detector (RID), as described by Dziekońska-Kubczak et al. [23].

Gas chromatographic analysis (HS–GC–MS) of the aldehydes in the fermented mashes was performed using a GC apparatus (Agilent 7890A, Agilent Technologies, Santa Clara, CA, USA) coupled to a mass spectrometer (Agilent MSD 5975C, Agilent Technologies, Santa Clara, CA, USA). A capillary column was used to separate the compounds (VF-WaxMS, Agilent, USA; 60 m × 0.32 mm × 0.50 μm). The GC oven temperature was programmed to increase from 30 °C (6 min) to 220 °C at a rate of 10 °C/min, where it was maintained for 5 min. The flow rate of the carrier gas (helium) through the column was 1.1 mL/min. The temperature of the injector (split/splitless) was 250 °C. Injections of the tested samples were made in the split mode (25:1) using a headspace analyser (Agilent 7697A, Agilent Technologies, Santa Clara, CA, USA). The temperatures of the MS ion source, transfer line, and quadrupole were 230, 250, and 150 °C, respectively. The ionization energy was 70 eV. Prior to analysis, a 20 mL headspace vial was filled with 7 mL of mash and closed tightly using an aluminium cap and septa.

Headspace conditions:

- Temperature settings: oven temperature 50 °C, loop temperature 60 °C, transfer line temperature 70 °C.
- Timing settings: vial equilibration time 20 min, injection duration 0.7 min, GC cycle time 47 min.
- Vial and loop settings: vial shaking 71 shakes/min, fill pressure 15 psi, vial pressurization gas helium.

### 2.5. Calculations

The theoretical fermentation efficiency was calculated in relation to total sugars, according to the stoichiometric Gay-Lussac’s equation. The real efficiency was calculated taking into consideration the ethanol concentration in the fermented mashes and expressed as a percentage of the theoretical value.

### 2.6. Statistical Analysis

All experiments were performed in triplicate. In order to verify whether the applied variables (i.e., yeast strain, pH of the sweet mash, and the method of starch liberation) affected the concentration of aldehydes in the fermented mash, the results from the GC–MS analysis were statistically analysed using an ANOVA, with a significance level of 0.05. If significant differences were found, Tukey**’**s multiple comparison test (post hoc test) was performed.

The calculated fermentation efficiency (separately for each of the mash preparation method) was also subjected to a statistical analysis using an ANOVA, at a significance level of 0.05, followed by Tukey**’**s post hoc test. All calculations were performed using Statistica 13.3.1 (TIBCO Software Inc., Palo Alto, CA, USA).

## 3. Results and Discussion

### 3.1. Chemical Composition of Distillery Mashes before Fermentation

The chemical composition of distillery mashes depends mainly on the type of raw materials, processing methods, and fermentation conditions [24]. In this study, rye-grain-based sweet mashes were prepared using two methods: the pressure-thermal method known as steaming and the pressureless method (PLS). The mashes were analysed in terms of sugars and acid concentrations. The results are shown in Table 1. The extract content of the sweet mashes ranged between (163.22 ± 2.55) g/kg for the mashes prepared by the pressure-thermal method and (182.53 ± 1.54) g/kg for the mashes prepared by the PLS method. The initial treatment of rye grains had a significant effect on the concentration of individual sugars after the saccharification of the starch. The fermentable sugars present in the mash prepared from the raw material after the pressure-thermal treatment, before the hydrolysis of dextrins, consisted of glucose at a concentration of (67.06 ± 3.35) g/L, maltose at a concentration of (15.89 ± 0.79) g/L, and maltotriose at a concentration of (10.57 ± 0.53) g/L. The mashes prepared using the PLS method were characterised by a much higher concentration of maltose (72.70 ± 3.64) g/L and lower concentrations of glucose (35.91 ± 1.80) g/L and maltotriose (7.79 ± 0.39) g/L, compared to the mash obtained by the steaming method. The differences in the quantitative compositions of fermentable sugars in the mashes, depending on the method of preparation, result from the fact that the gelatinisation of starch occurs during pressure-thermal treatment, promoting a deeper starch hydrolysis [24].

An acid hydrolysis of the dextrins was conducted to calculate the total concentration of sugars. Based on the results of the HPLC analysis (Table 1), the glucose content about doubled in the mash prepared from steamed raw material and increased more than fourfold in the mashes made using the pressureless method. The concentrations of maltotriose and maltose decreased, as a result of the hydrolysis to glucose. The concentrations of total sugars, calculated on the basis of the glucose, maltose, and maltotriose concentrations and expressed as glucose, ranged from (147.68 ± 7.38) g/L of the mash from the steamed rye grain to (165.27 ± 8.26) g/L of the mash from the PLS method. The differences we observed, despite using the same proportions of raw material and water in both methods, were most probably the consequence of the dilution of the steamed mass with condensate that formed after the completion of steaming.

Apart from fermentable sugars, the mashes contained xylose, arabinose, and acetic acid as products of the hemicellulose hydrolysis, as well as formic acid as a product of the glucose decomposition during the thermal treatment of the raw material [25]. Significantly higher amounts of these compounds (except for acetic acid) were present in the mash prepared by the pressure-thermal treatment method (Table 1).

### 3.2. Characteristics of Distillery Mashes after Fermentation

The analysis of the chemical compositions of the fermented mashes (Table 2 and Table 3) showed that in the mashes prepared from steamed raw material, maltotriose was present at concentrations from (0.018 ± 0.002) to (0.092 ± 0.015) g/L only in the samples with an initial pH of between 5.0 and 6.0, fermented using the Ethanol Red yeast strain. All samples of mashes prepared using the PLS method, regardless of the yeast strain used for the fermentation, contained significantly higher concentrations of maltotriose, from (1.485 ± 0.022) g/L (pH 6.0, DistillaMax GW) to (2.148 ± 0.075) g/L (pH 6.0, Ethanol Red). These samples also contained significantly higher amounts of maltose, from (0.870 ± 0.018) g/L to (1.748 ± 0.348) g/L, and glucose, from (1.666 ± 0.162) g/L to (11.794 ± 0.520) g/L, compared to the analogous samples with the pressure-thermal treatment, the majority of which contained these sugars in amounts far below 0.5 g/L. The differences in the utilisation of fermentable sugars indicate that the liquefaction of the starch during the pressure-thermal treatment facilitates its hydrolysis during fermentation, a finding confirmed by our previous study [26]. All the fermented mashes, produced using both the pressure-thermal and pressureless methods, contained much higher concentrations of pentose sugars than the sweet mashes. This was a consequence of the action of a supportive enzyme preparation (Viscozyme), which was added to lower the viscosity of the mashes. Unfortunately, *S. cerevisiae* does not naturally utilise xylose and arabinose, because of the lack of an upstream module of the appropriate metabolic pathway [27]. Moreover, all mashes after fermentation completion contained glycerol, which is a by-product of yeast metabolism [28]. The concentrations of glycerol were, on average, approximately 50% higher in the samples prepared using the PLS method than in those from the steamed raw material. Among environmental factors affecting glycerol content are the sugar content, the temperature, and the pH. Higher pH values enhance the glycerol yield in the fermentation medium [28], which was reflected in the results of our study. Lower amounts of glycerol were produced by DistilaMax GW than by the DistilaMax HT and Ethanol Red yeast strains (Table 2 and Table 3). The activities of enzymes involved in glycerol production are correlated to the genetic profile of yeast strains [29], which may explain the observed differences in its concentrations in the tested rye mashes.

The mashes after fermentation contained much lower concentrations of acetic acid than the sweet mashes. Acetic acid may be formed by *Saccharomyces cerevisiae* as a normal by-product of alcoholic fermentation, with additional amounts being produced by lactic acid bacteria and/or acetic acid bacteria. The concentration of acetic acid produced during alcoholic fermentation may vary with the species and strain of yeast. Acetic acid produced by *S. cerevisiae* forms rapidly during fermentation, but some is later metabolised [30], which is what probably took place in our study. The fermentation of rye mashes prepared with both pressure-thermal and pressureless treatments, with an initial pH of between 4.5 and 6.0, using the DistilaMax HT yeast strain, resulted in ethanol concentrations of between (62.93 ± 2.13) and (65.27 ± 1.63) g/L (*p* > 0.05). The use of DistilaMax GW for the fermentation of the mashes led to the highest ethanol concentrations. In the mashes obtained using the pressure-thermal method, the ethanol concentrations ranged from (63.57 ± 2.17) to (66.20 ± 0.24) g/L. The samples of mashes prepared by the PLS method contained ethanol concentrations of between (67.19 ± 2.74) g/L (pH 4.5) and (69.18 ± 0.76) g/L (pH 5.0), (*p* > 0.05). As a consequence of efficient ethanol production, these samples showed the highest utilisation of sugars. The lowest ethanol contents were produced by the Ethanol Red yeast strain, especially in the mashes prepared using the pressureless method. The ethanol contents in these mashes were between (56.33 ± 2.44) and (58.92 ± 1.35) g/L, regardless of the initial pH of the medium. These samples also showed the highest concentrations of glucose after fermentation, which may indicate lower fermentative activity of Ethanol Red compared to the other yeast strains. The knowledge of the genotypes of the tested yeast strains would be helpful to assess in depth the fermentation abilities of the tested yeast strains. 

### 3.3. Fermentation Efficiency

In order to evaluate the fermentation results, the efficiency of the ethanol biosynthesis (expressed as a percentage of the theoretical amount) was calculated according to the stoichiometric equation of Gay-Lussac. The fermentation efficiency (actual yield relative to the theoretical) reached higher values in the case of mashes prepared using the pressure-thermal treatment, from 82.33% to 87.71% of the theoretical yield, compared to that for the mashes prepared by the PLS method, which ranged from 66.68% to 81.89% of the theoretical yield (Table 4). An analysis of variance showed that the yeast strains influenced the fermentation efficiency. A further analysis using Tukey’s post hoc test confirmed that in the case of the mashes obtained after pressure-thermal treatment, only two samples showed statistical differences. The sample fermented with DistilaMax GW (initial pH 5.0) achieved the highest fermentation efficiency (87.71% of the theoretical one). The sample fermented with the Ethanol Red yeast (initial pH 6.0) showed the lowest fermentation efficiency (82.33% of the theoretical one). In the case of the mashes prepared using the PLS method, the calculations showed that the fermentation efficiency was higher when DistilaMax GW was used than with Ethanol Red or DistilaMax HT yeast strains. In the case of DistilaMax HT yeast, the differences concerned samples with an initial pH of between 5.0 and 6.0. However, there was no strong correlation between pH and fermentation efficiency (*p* > 0.05). It can therefore be concluded that the yeast strains used may perform efficient fermentation in the pH range from 4.5 to 6.0. Liu et al. [31] studied the effect of the initial pH on the growth and fermentation properties of *Saccharomyces cerevisiae* yeast. Their results showed that the initial pH of the fermentation medium was a vital factor influencing yeast growth and alcoholic fermentation. The maximum ethanol concentration observed for the majority of the tested strains was at pH 4.50.

### 3.4. Aldehydes in the Sweet and Fermented Rye Mashes

The factors determining the quality of agricultural distillates obtained from starchy raw materials include: the type and quality of the raw material; the method of mash preparation; the yeast strain and dose; and the fermentation and distillation conditions [26]. Carbonyls are the main volatile compounds occurring in rye grain [32]. The following aldehydes were determined in the sweet rye mashes (before fermentation): acetaldehyde, propionaldehyde, isobutyraldehyde, 2-methylbutyraldehyde, isovaleraldehyde, valeraldehyde, capronaldehyde, enanthaldehyde, pelargonaldehyde (Table 5). Acetaldehyde was the dominant compound in the mashes treated using both the pressure-thermal and pressureless methods. Acetaldehyde can result from the decomposition of sugars during the heating of plant raw materials [33]. Its concentration in the mash from steamed rye grain was more than double that in the mash prepared by the PLS method. Moreover, higher concentrations of isobutyraldehyde, 2-methylbutyraldehyde, isovaleraldehyde, and pelargonaldehyde were found in the sweet mash obtained by the pressure-thermal method compared to the mash obtained by the PLS method. The biosynthesis of isobutyraldehyde (2-methyl propanal) is generally associated with valine catabolism and is a result of the chemical oxidation of α-keto-isocaproic acid, catalysed by manganese [34]. An important process leading to the formation of aldehydes such as isovaleraldehyde (3-methylbutanal) is the nonenzymic, heat-induced, Strecker degradation of amino groups with reducing sugar moieties [35]. This aldehyde may be an intermediate in the catabolism of leucine [36]. Heptanal, known as enanthaldehyde, is a fatty aldehyde resulting from membrane lipid oxidation and may originate from rye grain [37]. Pelargonaldehyde (nonanal) is a volatile organic compound found in plants, including rye grain [38,39].

The analysis of the content of aldehydes in the fermented mashes (Table 6 and Table 7) showed that acetaldehyde was the dominant compound. Acetaldehyde occurs as an indirect metabolite of the transformation of sugars into ethyl alcohol during the alcoholic fermentation of various plant raw materials [7,8]. In the mashes from steamed rye grain, the concentrations of acetaldehyde varied between (14.343 ± 1.779) mg/L (DistilaMax HT, pH 4.5) and (253.941 ± 7.953) mg/L (DistilaMax GW, pH 6.0). In the mashes prepared using the PLS method, its concentrations varied between (26.795 ± 5.850) mg/L (DistilaMax HT, pH 5.0) and (67.744 ± 5.221) mg/L (DistilaMax GW, pH 6.0). Increasing the initial pH of the mashes from 4.5 to 6.0 caused a significant increase in acetaldehyde concentration, regardless of the yeast strains used, to a maximum in the sample fermented with DistilaMax GW (Table 6). An analogous relation was observed in the samples prepared by the PLS method. However, increasing the pH of these mashes did not cause such a large increase in acetaldehyde concentration as the one that occurred in the samples prepared from steamed rye grain. Interestingly, the highest acetaldehyde content was also determined in the sample fermented with DistilaMax GW yeast. The obtained results are in line with the literature data that the acetaldehyde production during ethanol fermentation is strain specific [40], and an increase in the pH of the fermentation medium results in an elevated acetaldehyde production [14]. Several studies have suggested to investigate yeast acetaldehyde production and to include this trait among yeast strain selection parameters [40]. The accumulation of acetaldehyde during fermentation is reported to be not related to the activity of the enzymes**’** alcohol and aldehyde dehydrogenase but probably to the equilibrium between their oxidised and reduced coenzymes and to the rate of uptake of reducing sugars and acetate [41]. Romano et al. [40] distinguished different yeast phenotypes: low, medium, and high acetaldehyde producers. The low and high phenotypes also differed considerably in the production of acetic acid, acetoin, and higher alcohols and can be useful for studying acetaldehyde production in *S. cerevisiae*, both from the technological and genetic point of views.

The majority of the tested rye mashes, prepared by the pressure-thermal treatment (Table 6), contained significantly higher amounts of acetaldehyde than the samples prepared by the PLS method with the same pH and fermented with the same yeast (Table 7). The only exception was in the case of mashes with an initial pH of 4.5, which contained significantly lower amounts of acetaldehyde than the analogous samples with the pressureless pretreatment. This result was confirmed by Tukey’s post hoc test. The higher concentrations of acetaldehyde in the majority of mashes from rye grain after the pressure-thermal pretreatment may be a consequence of the inhibiting effect of Maillard reaction products on the activity of alcohol dehydrogenase, which catalysed the reduction of acetaldehyde to ethanol [12]. The samples of mash fermented with DistilaMax HT (except for the sample from steamed raw material with an initial pH of 6.0) were characterised by lower concentrations of acetaldehyde, compared to those fermented with the other tested yeast strains. The highest concentrations of this acetaldehyde were determined in the samples fermented by DistilaMax GW, regardless of the method of mash preparation.

The concentrations of all other aldehydes present in the fermented mashes were significantly lower than the concentrations of acetaldehyde and were differentiated according to both the initial pH of the mash and the yeast used. Increasing the pH of the sweet mashes caused an increase in the concentrations of aldehydes from C3 (propionaldehyde) to C5 (valer- and isovaleraldehyde) in the samples after both pressure-thermal and pressureless treatments. Their share in the total content of aldehydes in the mashes from steamed rye grains ranged from 0.5% (Ethanol-Red, pH 6.0) to approximately 3.5% (DistilaMax HT, pH 4.5). Their share was higher in the samples prepared using the PLS method, from approximately 2.5% (DistilaMax HT, pH 4.5) to approximately 15% (DistilaMax HT, pH 5.5). This substantial difference may be explained to a significant extent by the large differences in the concentrations of acetaldehyde and capronaldehyde determined in the mashes prepared by the pressureless method, compared to the samples prepared by the pressure-thermal method. Caprylaldehyde (octanal) is a product of the degradation of lipids present in cereal grains [42] and was probably degraded during the steaming of the rye grain.

Since the content of aldehydes, especially acetaldehyde, is subject to limitations in agricultural distillates, we attempted to identify the optimal conditions for obtaining distillates with low concentrations of aldehydes. To this end, we conducted a statistical analysis of the influence of the examined variables and their interactions on the concentration of aldehydes (Table 8).

All the individually tested variables as well as their interactions were found to have significant effects on the concentrations of the majority of the determined aldehydes. Based on our analysis of the effects of the interaction of the method of mash preparation × pH of the sweet mash on the dominant compound (acetaldehyde), it can be concluded that the initial pH of mashes prepared using the pressure-thermal method should be adjusted to 4.5. This enables a high fermentation efficiency (Table 4) and results in a distillate with the lowest acetaldehyde content. Increasing the pH of the mash before fermentation to pH 5.0, 5.5, or 6.0 may result in significantly higher acetaldehyde concentrations. In the case of a mash prepared by the PLS method, pH levels in the range of 4.5 to 5.5 do not significantly affect the concentration of acetaldehyde, but further increasing the pH is not recommended because it may lead to an increase in the concentration of acetaldehyde (Table 8).

The analysis of the interaction of method of mash preparation × pH of sweet mash × yeast strain showed that to limit acetaldehyde production, the initial pH of the sweet mash prepared using the pressure-thermal method should be adjusted to 4.5. The recommended yeast strain is DistilaMax HT. In the case of the fermentation of steamed rye mashes with the other yeast strains (DistilaMax GW and Ethanol Red), the initiation of fermentation at pH 4.5 is also recommended, since the concentration of acetaldehyde was significantly higher in the samples with a higher pH. In the case of mashes prepared by the PLS method, DistilaMax HT yeast produced lower concentrations of aldehydes at pH levels in the range of 4.5–5.5. It is definitely not recommended to ferment sweet mash with an initial pH of 6.0; this concerns mashes prepared by both methods of pretreatment used in this study.

## 4. Conclusions

Aldehydes are fermentation by-products, which may worsen the quality of agricultural distillates and increase the costs of further purification. This study investigated the effects of two methods of rye mash preparation (pressure-thermal and pressureless) on the fermentation yield and concentrations of aldehydes in fermented rye mashes. It also investigated the effects of the initial pH of the sweet mash (4.5, 5.0, 5.5, and 6.0) and of different yeast strains (DistilaMax GW, DistilaMax HT, and Ethanol Red). The results revealed that the tested factors, i.e., the method of mash preparation, initial pH of the sweet mash, and yeast strain, both individually as well as their interactions, had a significant influence on both the fermentation efficiency and the concentrations of aldehydes—especially acetaldehyde—which on average accounted for approximately 90% of all the aldehydes determined in the tested rye mashes.

Mashes prepared from steamed rye grain allowed for a higher fermentation efficiency. However, these mashes contained significantly higher concentrations of acetaldehyde than those prepared by the pressureless method. Increasing the pH of the sweet mashes from 4.5 to 6.0 resulted in increased concentrations of acetaldehyde in the samples after both pressure-thermal and pressureless treatments. Adjusting the pH of the mashes to 4.5 was found to be optimal for obtaining a high fermentation efficiency and limiting the acetaldehyde biosynthesis, for both methods of mash preparation. The highest fermentation efficiency was obtained using the yeast strains DistilaMax GW and DistilaMax HT. The lowest concentrations of acetaldehyde were obtained with the use of DistilaMax HT. This promising yeast strain was able to provide not only a high fermentation efficiency but also a relatively low concentration of acetaldehyde in mashes with an initial pH in the range of 4.5–5.5 prepared by the energy-saving PLS method.

## Figures and Tables

**Table 1:** Chemical composition of sweet mashes obtained by the pressure-thermal method and pressureless (PLS) method.

Compound	Content (g/L)	Pressure-Thermal Method	PLS Method
Sugars before hydrolysis of dextrins			
Maltotriose	M	10.57 a	7.79 b
	SD	0.53	0.39
Maltose	M	15.89 b	72.70 a
	SD	0.79	3.64
Glucose	M	67.06 a	35.91 b
	SD	3.35	1.80
Sugars after hydrolysis of dextrins			
Maltotriose	M	1.01 b	1.25 a
	SD	0.05	0.06
Maltose	M	7.37 b	9.36 a
	SD	0.37	0.47
Glucose	M	138.84 b	154.08 a
	SD	6.94	7.70
Total sugars *	M	147.68 b	165.27 a
	SD	7.38	8.26
Other compounds			
Xylose	M	4.12 a	1.61 b
	SD	0.21	0.08
Arabinose	M	0.31 a	0.12 b
	SD	0.02	0.01
Formic acid	M	0.09 a	0.07 b
	SD	0.01	0.00
Acetic acid	M	0.24 a	0.25 a
	SD	0.01	0.01

M—mean value; SD—standard deviation (*n* = 3); * sum of fermentable sugars, expressed as glucose, after acid hydrolysis of dextrins; a-b—mean values in lines with different letters are significantly different (two-way ANOVA, *p* < 0.05).

**Table 2:** Chemical composition of mashes prepared by the pressure-thermal method and fermented by various yeast strains.

Compound	Content (g/L)	DistilaMax HT				DistilaMax GW				Ethanol Red			
		pH 4.5	pH 5.0	pH 5.5	pH 6.0	pH 4.5	pH 5.0	pH 5.5	pH 6.0	pH 4.5	pH 5.0	pH 5.5	pH 6.0
Maltotriose	M	n.d.	n.d.	n.d.	n.d.	n.d.	n.d.	n.d.	n.d.	n.d.	0.018 c	0.044 b	0.092 a
	SD	-	-	-	-	-	-	-	-	-	0.002	0.008	0.015
Maltose	M	0.314 e	0.392 c	0.328 e	0.304 f	0.399 c	0.450 b	0.408 c	0.317 f	0.500 a	0.366 d	0.160 g	0.062 h
	SD	0.013	0.020	0.007	0.012	0.004	0.015	0.005	0.011	0.008	0.023	0.003	0.002
Glucose	M	0.047 ef	0.054 e	0.055 e	0.068 e	0.018 g	0.019 g	0.021 g	0.032 f	0.390 d	0.424 c	0.479 b	0.619 a
	SD	0.016	0.008	0.015	0.010	0.004	0.007	0.007	0.007	0.012	0.013	0.009	0.014
Xylose	M	0.278 e	0.380 c	0.370 c	0.397 c	0.229 f	0.576 b	0.765 a	0.518 b	0.267 e	0.275 de	0.298 d	0.291 d
	SD	0.021	0.016	0.009	0.043	0.020	0.079	0.078	0.053	0.008	0.019	0.006	0.003
Arabinose	M	0.247 b	0.266 b	0.219	0.295 a	0.213	0.237	0.227 c	0.282 a	0.227	0.215	0.213	0.152
	SD	0.047	0.011	0.028	0.037	0.001	0.032	0.035	0.006	0.003	0.007	0.004	0.106
Glycerol	M	3.928 c	4.070 b	4.103 b	4.300 a	3.362 e	3.530 d	3.605 d	3.644 d	3.923 c	3.992 c	4.074 b	4.170 b
	SD	0.027	0.017	0.054	0.010	0.093	0.027	0.035	0.080	0.068	0.060	0.112	0.094
Acetic acid	M	0.066 e	0.070 de	0.067 e	0.079 d	0.055 f	0.046 g	0.028 h	0.061 e	0.107 c	0.110 abc	0.118 b	0.135 a
	SD	0.003	0.005	0.002	0.004	0.003	0.002	0.001	0.007	0.004	0.008	0.003	0.004
Ethanol	M	64.09 b	64.58 b	62.93 bc	63.99 b	64.35 ab	66.20 a	64.95 b	63.57 b	63.20 c	63.08 c	63.08 c	62.14 c
	SD	0.04	0.46	2.13	0.36	1.69	0.24	0.46	2.17	0.64	0.70	0.83	1.05

M—mean value; SD—standard deviation (n = 3); n.d.—not detected; Mean values with different letters (a, b, c, etc.) within the same lines are significantly different (two-way ANOVA, *p* < 0.05).

**Table 3:** Chemical composition of mashes prepared by the pressureless starch liberation method and fermented by various yeast strains.

Compound	Content (g/L)	DistilaMax HT				DistilaMax GW				Ethanol Red			
		pH 4.5	pH 5.0	pH 5.5	pH 6.0	pH 4.5	pH 5.0	pH 5.5	pH 6.0	pH 4.5	pH 5.0	pH 5.5	pH 6.0
Maltotriose	M	1.936 b	1.948 b	1.985 ab	1.987 ab	1.549 c	1.565 c	1.503 c	1.485 c	2.020 a	2.012 a	2.111 a	2.148 a
	SD	0.044	0.023	0.067	0.007	0.046	0.085	0.032	0.022	0.109	0.102	0.024	0.075
Maltose	M	1.066 bc	1.039 b	0.985 c	0.917 d	0.947 cd	0.927 d	0.895 e	0.870 e	1.579 a	1.533 a	1.748 a	1.547 a
	SD	0.110	0.034	0.012	0.020	0.031	0.022	0.009	0.018	0.157	0.128	0.348	0.163
Glucose	M	4.117 b	4.479 b	4.646 b	4.673 b	1.809 c	1.847 c	1.729 c	1.666 c	10.989 a	11.402 a	11.406 a	11.794 a
	SD	0.386	0.330	0.515	0.194	0.118	0.370	0.169	0.162	0.646	0.682	0.537	0.520
Xylose	M	0.984 b	1.082 b	1.210 a	1.285 a	0.681 d	0.859 c	0.859 c	0.878 c	0.663 d	0.685 d	0.485 e	0.469 e
	SD	0.099	0.038	0.040	0.102	0.020	0.037	0.034	0.033	0.101	0.105	0.011	0.016
Arabinose	M	0.076 d	0.093 c	0.099 c	0.096 c	0.102 bc	0.114 bc	0.128 a	0.132 a	0.101 bc	0.112 bc	0.105 bc	0.099 bc
	SD	0.005	0.002	0.011	0.008	0.019	0.012	0.003	0.004	0.007	0.024	0.018	0.007
Glycerol	M	6.133 b	6.234 ab	6.494 a	6.589 a	5.100 e	5.481 d	5.698 c	5.888 c	5.787 c	5.766 c	6.175 b	6.555 a
	SD	0.157	0.243	0.196	0.232	0.190	0.058	0.033	0.018	0.140	0.335	0.175	0.025
Acetic acid	M	0.027 cd	0.029 cd	0.054 b	0.074 a	0.037 c	0.028 d	0.034 cd	0.032 cd	0.025 d	0.023 d	0.031 cd	0.028 cd
	SD	0.005	0.006	0.009	0.003	0.006	0.001	0.008	0.008	0.003	0.004	0.005	0.004
Ethanol	M	65.27 b	64.03 b	64.03 b	63.92 b	67.19 a	69.18 a	69.15 a	69.13 a	58.92 c	56.33 c	57.04 c	58.09 c
	SD	1.63	1.12	1.05	0.88	2.74	0.76	0.77	1.63	1.35	2.44	0.61	0.62

M—mean value; SD—standard deviation (n = 3); Mean values with different letters (a, b, c, etc.) within the same lines are significantly different (two-way ANOVA, *p* < 0.05).

**Table 4:** Fermentation efficiency of rye mashes.

Yeast Strain	Initial pH of the Mash	Fermentation Efficiency (% of the Theoretical Value)	
		Pressure-Thermal Method	PLS Method
DistilaMax HT	pH 4.5	84.88 ab	77.50 ab
	pH 5.0	85.56 ab	75.79 a
	pH 5.5	83.38 ab	75.80 a
	pH 6.0	84.78 ab	75.67 a
DistilaMax GW	pH 4.5	85.26 ab	79.55 ab
	pH 5.0	87.71 b	81.89 b
	pH 5.5	86.05 ab	81.86 b
	pH 6.0	84.22 ab	81.84 b
Ethanol Red	pH 4.5	83.73 ab	69.75 c
	pH 5.0	83.58 ab	66.68 c
	pH 5.5	83.57 ab	67.52 c
	pH 6.0	82.33 a	68.77 c

a–c—Mean values in columns with different letters are significantly different (two-way ANOVA, *p* < 0.05).

**Table 5:** Aldehydes in the sweet rye mashes.

Compound	Concentration (mg/L of Mash)		
	M	Pressure-Thermal Method	PLS Method
	SD		
Acetaldehyde	M	1.952 a	0.832 b
	SD	0.098	0.040
Propionaldehyde	M	0.011 a	0.012 a
	SD	0.001	0.001
Isobutyraldehyde	M	0.078 a	0.024 b
	SD	0.004	0.002
2-Methylbutyraldehyde	M	0.022 a	0.014 b
	SD	0.001	0.001
Isovaleraldehyde	M	0.051 a	0.029 b
	SD	0.003	0.002
Valeraldehyde	M	0.011 a	0.017 a
	SD	0.005	0.001
Capronaldehyde	M	0.260 b	0.456 a
	SD	0.013	0.020
Enanthaldehyde	M	0.005 a	0.005 a
	SD	0.000	0.001
Pelargonaldehyde	M	0.042 a	n.d.
	SD	0.002	-

M—mean value; SD—standard deviation (n = 3); n.d.—not detected; mean values with different letters (a, b) within the same lines are significantly different (two-way ANOVA, *p* < 0.05).

**Table 6:** Concentrations of aldehydes in the fermented mashes prepared using the pressure-thermal method.

Compound	Concentration (mg/L)													Results of Two-Way ANOVA		
	M	DistilaMax HT				DistilaMax GW				Ethanol Red						
	SD	pH 4.5	pH 5.0	pH 5.5	pH 6.0	pH 4.5	pH 5.0	pH 5.5	pH 6.0	pH 4.5	pH 5.0	pH 5.5	pH 6.0	YS	IpH	YS × IpH
Acetaldehyde	M	14.343 i	34.464 gh	105.37 d	216.819 b	23.166 hi	52.111 g	146.973 f	253.941 a	28.160 hi	77.097 e	145.143 f	191.304 c	***	***	***
	SD	1.779	1.621	10.728	3.278	7.326	6.078	3.520	7.953	3.943	8.653	8.429	8.864			
Propionaldehyde	M	0.011 f	0.016 f	0.034 d	0.070 b	0.014 f	0.019 ef	0.038 cd	0.100 a	0.014 f	0.024 e	0.043 c	0.064 b	***	***	***
	SD	0.001	0.001	0.004	0.003	0.012	0.002	0.003	0.002	0.001	0.002	0.003	0.004			
Isobutyraldehyde	M	0.083 gh	0.127 gh	0.294 ef	0.518 b	0.048 h	0.088 gh	0.319 e	0.840 a	0.150 dg	0.223 df	0.299 ef	0.402 c	***	***	***
	SD	0.011	0.009	0.045	0.046	0.111	0.001	0.003	0.009	0.005	0.020	0.013	0.011			
2-Methylbutyr- aldehyde	M	0.035 d	0.065 cd	0.216 b	0.240 b	0.064 cd	0.073 cd	0.169 bc	0.749 a	0.038 d	0.050 d	0.048 d	0.085 cd	***	***	***
	SD	0.018	0.004	0.036	0.091	0.068	0.010	0.022	0.012	0.004	0.004	0.007	0.003			
Isovaleraldehyde	M	0.081 c	0.122 c	0.245 b	0.296 b	0.081 c	0.092 c	0.124 c	0.403 a	0.076 c	0.092 c	0.125 c	0.249 b	***	***	***
	SD	0.021	0.003	0.028	0.082	0.065	0.004	0.021	0.009	0.005	0.012	0.014	0.012			
Valeraldehyde	M	0.013 a	0.007 d	0.005 d	n.d.	0.013 a	0.009 bc	0.009 bc	0.009 bc	0.011 ab	n.d.	n.d.	n.d.	***	***	***
	SD	0.001	0.001	0.001	-	0.002	0.002	0.001	0.001	0.001	-	-	-			
Capronaldehyde	M	0.263 ef	0.214 f	0.437 c	0.401 cd	0.376 cd	0.337 cde	0.259 ef	0.247 ef	0.311 def	0.246 ef	0.122 ab	0.108 b	***	**	***
	SD	0.038	0.023	0.060	0.068	0.093	0.030	0.044	0.019	0.024	0.011	0.021	0.007			
Enanthaldehyde	M	0.021 d	0.021 cd	0.020 d	0.021 cd	0.025 ac	0.026 a	0.024 acd	0.024 acd	0.010 b	0.012 b	0.011 b	0.012 b	***	n.s.	n.s.
	SD	0.001	0.001	0.001	0.002	0.001	0.001	0.003	0.001	0.000	0.001	0.001				

M—mean value; SD—standard deviation (n = 3); n.d.—not detected; mean values with different letters (a, b, c, etc.) within the same lines are significantly different (*p* < 0.05); YS—yeast strain; IpH—initial pH of the sweet mash; *** *p* < 0.001, ** *p* < 0.01, * *p* < 0.05, n.s.—not significant.

**Table 7:** Concentrations of aldehydes in the fermented mashes prepared using the PLS method.

Compound	Concentration (mg/L)													Results of Two-Way ANOVA		
	M	DistilaMax HT				DistilaMax GW				Ethanol Red						
	SD	pH 4.5	pH 5.0	pH 5.5	pH 6.0	pH 4.5	pH 5.0	pH 5.5	pH 6.0	pH 4.5	pH 5.0	pH 5.5	pH 6.0	YS	IpH	YS × IpH
Acetaldehyde	M	29.377 de	26.795 d	29.613 de	34.556 de	40.095 ce	39.623 cde	47.713 bc	67.744 a	37.239 cde	30.421 de	41.816 ce	55.017 ab	***	***	**
	SD	1.834	5.850	6.281	7.620	3.167	5.068	2.932	5.221	1.374	1.888	3.139	2.577			
Propionaldehyde	M	0.009 cd	0.008 d	0.009 cd	0.009 bcd	0.010 bcd	0.010 bcd	0.011 bc	0.012 b	0.010 bcd	0.009 cd	0.012 ab	0.014 a	***	***	**
	SD	0.001	0.001	0.001	0.001	0.000	0.001	0.001	0.001	0.000	0.000	0.001	0.001			
Isobutyraldehyde	M	0.063 d	0.063 d	0.068 cd	0.078 cd	0.043 d	0.043 d	0.050 d	0.104 c	0.064 d	0.077 cd	0.220 b	0.378 a	***	***	***
	SD	0.001	0.003	0.003	0.002	0.004	0.002	0.004	0.005	0.005	0.012	0.041	0.011			
2-Methylbutyr- aldehyde	M	0.074 a	0.074 a	0.070 a	0.074 a	0.029 c	0.032 c	0.027 c	0.054 b	0.041 b	0.041 b	0.058 b	0.066 b	***	***	***
	SD	0.003	0.004	0.001	0.004	0.003	0.008	0.004	0.004	0.003	0.001	0.013	0.009			
Isovaleraldehyde	M	0.096 ef	0.101 ef	0.105 cef	0.123 ce	0.047 f	0.052 f	0.053 f	0.107 ef	0.085 df	0.086 df	0.137 ab	0.160 a	***	***	***
	SD	0.006	0.002	0.002	0.009	0.002	0.008	0.006	0.004	0.006	0.003	0.032	0.016			
Valeraldehyde	M	0.007 bc	0.005 de	0.005 ae	0.005 de	0.006 cd	0.006 de	0.005 de	0.005 e	0.009 a	0.007 b	0.005 de	n.d.	n.s.	***	***
	SD	0.001	0.000	0.000	0.001	0.000	0.000	0.000	0.000	0.000	0.000	0.000	-			
Capronaldehyde	M	0.469 a	0.422 ab	0.414 ab	0.354 bc	0.293 cd	0.299 cd	0.216 f	0.226 ef	0.241 ef	0.201 f	0.106 g	0.095 g	***	***	*
	SD	0.020	0.035	0.034	0.046	0.034	0.011	0.011	0.020	0.027	0.018	0.024	0.010			
Enanthaldehyde	M	0.033 ab	0.035 a	0.037 a	0.038 a	0.022 ef	0.021 f	0.022 f	0.023 def	0.026 cdef	0.028 cde	0.029 cde	0.028 cde	***	*	n.s.
	SD	0.003	0.002	0.003	0.003	0.002	0.001	0.001	0.001	0.002	0.001	0.001	0.001			
Caprylaldehyde	M	0.017 d	1.448 c	4.476 a	4.232 a	4.167 a	4.049 a	3.979 a	3.884 b	3.868 b	3.702 b	3.621 b	3.711 b	***	**	***
	SD	0.001	2.483	0.221	0.123	2.462	0.130	0.248	0.055	0.082	0.048	0.120	0.103			

M—mean value; SD—standard deviation (n = 3); n.d.—not detected; mean values with different letters (a, b, c, etc.) within the same lines are significantly different (*p* < 0.05); YS—yeast strain; IpH—initial pH of the sweet mash; *** *p* < 0.001, ** *p* < 0.01, * *p* < 0.05, n.s.—not significant.

**Table 8:** Results of three-way ANOVA.

Compound	MSL	YS	IpH	MSL × YS	MSL × IpH	YS × IpH	MSL × YS × IpH
Acetaldehyde	***	***	***	*	***	***	***
Propionaldehyde	***	***	***	***	***	***	***
Isobutyraldehyde	***	***	***	***	***	***	***
2-Methylbutyraldehyde	***	***	***	***	***	***	***
Isovaleraldehyde	***	***	***	***	***	***	***
Valeraldehyde	***	***	***	***	***	***	***
Capronaldehyde	n.s.	***	***	***	*	***	***
Enanthaldehyde	***	***	*	***	*	n.s.	n.s.

MSL—method of starch liberation; YS—yeast strain; IpH—initial pH of the sweet mash. *** *p* < 0.001, ** *p* < 0.01, * *p* < 0.05, n. s.—not significant.

## Data Availability

Not applicable.

## References

[1] (2019). Regulation (EU) 2019/787 of the European Parliament and of the Council. Off. J. Eur. Union.

[2] Pielech-Przybylska K., Balcerek M., Nowak A., Wojtczak M., Czyżowska A., Dziekońska-Kubczak U., Patelski P. (2017). The effect of different starch liberation and saccharification methods on the microbial contaminations of distillery mashes, fermentation efficiency, and spirits quality. Molecules.

[3] Balcerek M., Pielech-Przybylska K., Strąk E., Patelski P., Dziekońska U. (2016). Comparison of fermentation results and quality of the agricultural distillates obtained by application of commercial amylolytic preparations and cereal malts. Eur. Food Res. Technol..

[4] Dymerski T., Gębicki J., Wardencki W., Namieśnik J. (2013). Quality evaluation of agricultural distillates using an electronic nose. Sensors.

[5] Baert J.J., De Clippeleer J., Hughes P.S., De Cooman L., Aerts G. (2012). On the origin of free and bound staling aldehydes in beer. J. Agric. Food Chem..

[6] Balcerek M. (2010). Carbonyl compounds in aronia spirits. Pol. J. Food Nutr. Sci..

[7] Pielech-Przybylska K., Balcerek M., Grumezescu A.M., Holban A.M. (2019). Alcoholic Beverages.

[8] Hazelwood L.A., Daran J.M., van Maris A.J., Pronk J.T., Dickinson J.R. (2008). The Ehrlich pathway for fusel alcohol production: A century of research on *Saccharomyces cerevisiae* metabolism. Appl. Environ. Microbiol..

[9] Vuralhan Z., Morais M.A., Tai S.L., Piper M.D.W., Pronk J.T. (2003). Identification and characterization of phenylpyruvate decarboxylase genes in *Saccharomyces cerevisiae*. Appl. Environ. Microbiol..

[10] Walker G.M. (2004). Metals in yeast fermentation processes. Adv Appl Microbiol..

[11] Walker G.M., Batt C.A. (2014). Encyclopedia of Food Microbiology.

[12] Kłosowski G., Mikulski D., Rolbiecka A., Czupryński B. (2017). Changes in the concentration of carbonyl compounds during the alcoholic fermentation process carried out with *Saccharomyces cerevisiae* yeast. Pol. J. Microbiol..

[13] Cheraiti N., Guezenec S., Salmon J.-M. (2010). Very early acetaldehyde production by industrial *Saccharomyces cerevisiae* strains: A new intrinsic character. Appl. Microbiol. Biotechnol..

[14] Li E., de Orduña R.M. (2011). Evaluation of acetaldehyde production and degradation potential of enological *Saccharomyces* and non-*Saccharomyces* yeast strains in a resting cell model system. J. Ind. Microbiol. Biotechnol..

[15] Moreno-Arribas M.V., Polo M.C. (2009). Wine Chemistry and Biochemistry.

[16] Kłosowski G., Mikulski D. (2010). The effect of raw material contamination with mycotoxins on the composition of alcoholic fermentation volatile by-products in raw spirits. Bioresour. Technol..

[17] Kokkinidou S., Peterson D.G. (2016). Identification of compounds that contribute to trigeminal burn in aqueous ethanol solutions. Food Chem..

[18] Cometto-Muñiz J.E., Cain W.S. (1993). Efficacy of volatile organic compounds in evoking nasal pungency and odor. Arch. Environ. Occup. Health.

[19] Cometto-Muñiz J.E., Abraham M.H. (2010). Odor detection by humans of lineal aliphatic aldehydes and helional as gauged by dose-response functions. Chem. Senses.

[20] IARC (1988). Chemical composition of alcoholic beverages, Additives and contaminants. IARC Monographs on the Evaluation of Carcinogenic Risk to Humans.

[21] (2002).

[22] Lane R.H., Helrich K. (1995). Official Methods of Analysis of the Association of Official Analytical Chemists.

[23] Dziekońska-Kubczak U., Berłowska J., Dziugan P., Patelski P., Pielech-Przybylska K., Balcerek M. (2018). Nitric acid pretreatment of jerusalem artichoke stalks for enzymatic saccharification and bioethanol production. Energies.

[24] Strąk E., Balcerek M. (2015). Industrial technologies used for the production of ethanol. Acta Sci. Pol. Technol. Biotech..

[25] Sjulander N., Kikas T. (2020). Origin, Impact and control of lignocellulosic inhibitors in bioethanol production—A Review. Energies.

[26] Balcerek M., Pielech-Przybylska K., Dziekońska-Kubczak U., Patelski P., Strąk E. (2016). Fermentation results and chemical composition of agricultural distillates obtained from rye and barley grains and the corresponding malts as a source of amylolytic enzymes and starch. Molecules.

[27] Wei F., Li M., Wang M., Li H., Li Z., Qin W., Wei T., Zhao J., Bao X. (2021). A C6/C5 co-fermenting *Saccharomyces cerevisiae* strain with the alleviation of antagonism between xylose utilization and robustness. GCB Bioenergy.

[28] Yalcin S.K., Ozbas Z.Y. (2008). Effects of pH and temperature on growth and glycerol production kinetics of two indigenous wine strains of *Saccharomyces cerevisiae* from Turkey. Braz. J. Microbiol..

[29] Pérez-Torrado R., Oliveira B.M., Zemančíková J., Sychrová H., Querol A. (2016). Alternative glycerol balance strategies among *Saccharomyces* species in response to winemaking stress. Front. Microbiol..

[30] Vasserot Y., Mornet F., Jeandet P. (2010). Acetic acid removal by *Saccharomyces cerevisiae* during fermentation in oenological conditions. Metabolic consequences. Food Chem..

[31] Liu X., Jia B., Sun X., Ai J., Wang L., Wang C., Zhao F., Zhan J., Huang W. (2015). Effect of initial pH on growth characteristics and fermentation properties of *Saccharomyces cerevisiae*. J. Food Sci..

[32] Heiniö R.L., Katina K., Wilhelmson A., Myllymäki O., Rajamäki T., Latva-Kala K., Liukkonen K.-H., Poutanen K. (2003). Relationship between sensory perception and flavour-active volatile compounds of germinated, sourdough fermented and native rye following the extrusion process. LWT—Food Sci. Technol..

[33] Rustemeier K., Stabbert R., Haussmann H.J., Roemer E., Carmines E.L. (2002). Evaluation of the potential effects of ingredients added to cigarettes. Part 2: Chemical composition of mainstream smoke. Food Chem. Toxicol..

[34] Smit B.A., Engels W.J., Alewijn M., Lommerse G.T., Kippersluijs E.A., Wouters J.T., Smit G. (2004). Chemical conversion of alpha-keto acids in relation to flavor formation in fermented foods. J. Agric. Food Chem..

[35] Strecker A. (1862). On a peculiar oxidation by alloxan. Justus Liebigs Ann. Chem..

[36] Smit B.A., Engels W.J.M., Smit G. (2009). Branched chain aldehydes: Production and breakdown pathways and relevance for flavour in foods. Appl. Microbiol. Biotechnol..

[37] The Metabolomic Innovations Centre. https://foodb.ca/compounds/FDB008048.

[38] Dudareva N., Klempien A., Muhlemann J.K., Kaplan I. (2013). Biosynthesis, function and metabolic engineering of plant volatile organic compounds. New Phytol..

[39] Buśko M., Jeleń H., Góral T., Chmielewski J., Stuper K., Szwajkowska-Michałek L., Tyrakowska B., Perkowski J. (2010). Volatile metabolites in various cereal grains. Food Addit. Contam. Part A.

[40] Romano P., Suzzi G., Turbanti L., Polsinelli M. (1994). Acetaldehyde production in *Saccharomyces cerevisiae* wine yeasts, *FEMS Microbiol*. Lett..

[41] Millán C., Ortega J.M. (1988). Production of ethanol, acetaldehyde, and acetic acid in wine by various yeast races: Role of alcohol and aldehyde dehydrogenase. Am. J. Enol. Vitic..

[42] Urban-Alandete L. (2019). Lipid Degradation during Grain Storage: Markers, Mechanisms and Shelf-Life Extension Treatments. Ph.D. Thesis.

